# Do gaze and non-gaze stimuli trigger different spatial interference effects? It depends on stimulus perceivability

**DOI:** 10.3389/fpsyg.2022.801151

**Published:** 2022-09-13

**Authors:** Zhe Chen, Rebecca H. Thomas, Makayla S. Chen

**Affiliations:** School of Psychology, Speech and Hearing, University of Canterbury, Christchurch, New Zealand

**Keywords:** gaze, arrows, stimulus perceivability, attentional zoom, spatial congruency effects

## Abstract

Among the studies on the perception of gaze vs. non-gaze stimuli, some have shown that the two types of stimuli trigger different patterns of attentional effects, while others have reported no such differences. In three experiments, we investigated the role of stimulus perceivability in spatial interference effects when the targets were gaze vs. non-gaze stimuli. We used a spatial Stroop task that required participants to make a speeded response to the direction indicated by the targets located on the left or right side of fixation. In different experiments, the targets consisted of eyes, symbols, and/or arrows. The results showed that the magnitude of the spatial congruency effect differed between the types of targets when stimulus perceivability was not controlled. However, when the perceivability of the task relevant parts was comparable between the different types of targets, similar congruency effects were found regardless of target type. These results underscore the importance of controlling for stimulus perceivability, which is closely linked to the attentional zoom required to perform a task, when making inferences about the attentional mechanisms in the processing of gaze vs. non-gaze stimuli.

## Introduction

Many studies have demonstrated that perceiving someone else’s gaze induces attention to orient toward the direction of the gaze, even when the direction indicated by the gaze is task irrelevant (Friesen and Kingstone, 1998; Langton and Bruce, 1999) or detrimental to the behavioral goal (Driver et al., 1999; Downing et al., 2004; Friesen et al., 2004). Given that our ability to interpret the gaze of others is vital for communicating with those around us, it seems plausible that the attentional mechanisms that underlie the processing of gaze may differ from those that underlie the processing of ordinary stimuli such as arrows (see Langton et al., 2000; Friesen et al., 2007, for reviews).

Consistent with this view is the finding that it takes longer to respond to gaze than to arrows (Hietanen et al., 2006; Marotta et al., 2018; see also Chacón-Candia et al., 2022 for a review). Furthermore, this pattern of data has been found only in typically developing individuals, but not in those with autism (Vlamings et al., 2005). As people with autism are known to have problems with social communication and social interaction (American Psychiatric Association, 2013), these results support the idea that the processing of gaze differs from the processing of non-gaze stimuli.

Gaze and non-gaze stimuli have also been shown to trigger different spatial interference effects. Using a spatial Stroop task (see Lu and Proctor, 1995, for a review), Marotta et al. (2018) required participants to make a speeded response to the direction indicated by either a pair of eyes or a pair of arrows on the left or right of fixation. Thus, the relationship between the direction indicated by the eyes/arrows and their location on the screen could be congruent (e.g., eyes looking left and on the left side of the screen) or incongruent (e.g., eyes looking left but on the right side of the screen). Different spatial congruency effects were found in the eye and arrow conditions. Whereas the average reaction time (RT) was shorter in the congruent than incongruent trials in the arrow condition, it was longer in the eye condition. Marotta et al. interpreted the inverse congruency effect in the eye condition in terms of the eyes being a special type of stimuli. In the incongruent trials, the gaze was directed toward the center, making the eyes appear to look at the participants. In the congruent trials, the eyes seemed to look away from the participants. According to the researchers, as it was more important to understand the meaning of the gaze when it was directed toward the center (i.e., the viewer) in the incongruent condition, processing of the gaze was more efficient.

However, there is also an alternative account. In Marotta et al. (2018), the eyes and arrows differed not only in their importance to social communication, but also in their perceivability, which was evidenced in the longer RTs in the eye condition than in the arrow condition. The perceivability of a stimulus, which is typically indicated by RT and/or accuracy, can be influenced by a variety of factors including salience, presentation duration, the size of the task relevant region, and distance from distractors. When the task relevant information of a target is difficult to perceive, focused attention is needed to encode the information, and this requires participants to adopt a small attentional zoom. With a small attentional zoom, task irrelevant information is more likely to be excluded (LaBerge et al., 1991; Chen and Cave, 2016). In Marotta et al., the directional information conveyed by the eyes was harder to perceive than that conveyed by the arrows, as the parts of the eyes that provided the critical information were smaller and less salient due to the surrounding flesh-colored eyelids. To discriminate the direction of the gaze, participants would need to use a relatively small attentional zoom so that attention could be focused on the parts that provided the critical information. Because the targets were presented against a large rectangle whose location on the screen was task irrelevant, the entire rectangle was unlikely to be encompassed within the focus of attention. As a result, the location of the rectangle on the screen, as well as that of the targets, was unlikely to be represented well. With the eyes and arrows presented in different blocks, participants probably deployed different extents of attentional zoom between the two conditions, resulting in different patterns of spatial interference effects.

In the three experiments reported here, we investigated the role of stimulus perceivability in spatial interference effects in a selective attention task when the targets were gaze vs. non-gaze stimuli. Understanding this question is important because this knowledge will inform us of the generality of the attentional mechanisms: whether the same system is involved in the processing of both gaze and non-gaze stimuli or whether there is a unique attentional system devoted to the processing of gaze due to its special role in social interaction. In all the experiments reported here, we used a spatial Stroop paradigm. We manipulated stimulus perceivability by using different types of gaze and non-gaze stimuli whose perceivability was either comparable or not comparable. In Experiment 1, the targets were either realistic-looking eyes (low perceivability) or arrows (high perceivability). In Experiment 2, the arrows were replaced by infinity symbols, which resembled the eyes in perceivability but differed from the eyes in the importance in social communication. In Experiment 3, we used cartoon eyes, nonsense symbols, and arrows in a between-subjects design while controlling for stimulus perceivability. To forecast our results, we found different patterns of spatial congruency effects only when the targets differed in perceivability. These results indicated that stimulus perceivability rather than social significance was the primary factor that gave rise to the observed results in the present paradigm.

## Experiment 1

**Figure 1:**
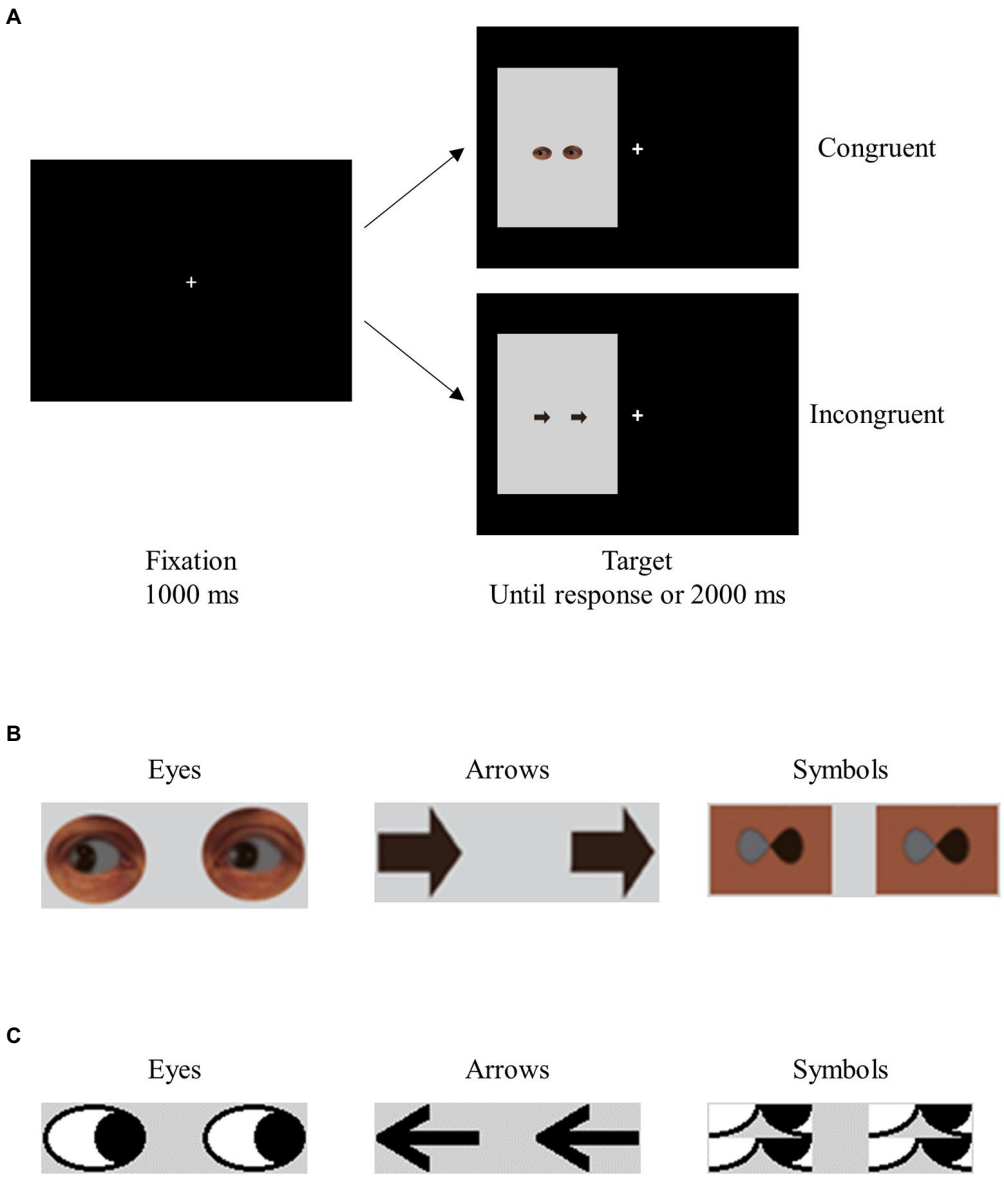
Sample trials in Experiment 1 **(A)**, and sample stimuli in Experiments 1 and 2 **(B)** and 3 **(C)**.

Experiment 1 was modeled after Marotta et al. (2018).^1^ Participants in different blocks saw two realistic-looking eyes gazing left or right, or two arrows pointing left or right. The task was to make a speeded response to the direction indicated by the target stimuli (see Supplementary material for a detailed description of the method). The targets, which pointed left or right, were on the left or right of fixation (see Figures 1A,B). The location of the targets and the direction they indicated were equally likely to be congruent (e.g., a target pointing left was on the left side of the screen) and incongruent (e.g., a target pointing left was on the right side of the screen). Thus, the experiment used a 2 × 2 repeated-measures design, with TargetType (eye vs. arrow) and Congruency (congruent vs. incongruent) as the principal factors.

### Results and discussion

**Figure 2:**
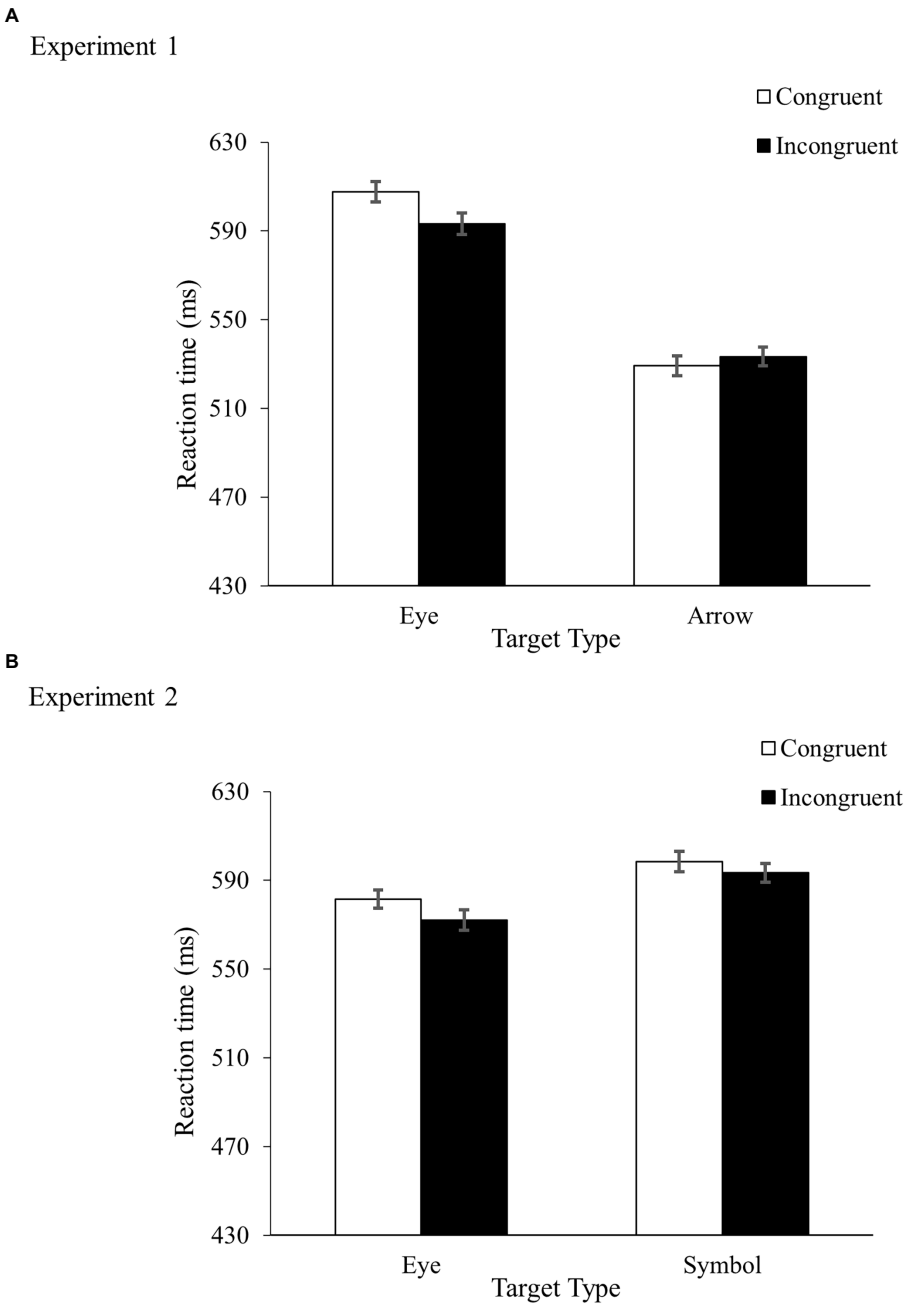
Results from Experiment 1 **(A)** and Experiment 2 **(B)**. Figures show mean RTs with error bars indicating plus and minus one within-subjects standard error of the mean.

Table 1 shows the error rates and Figure 2A shows the mean RTs. A repeated-measures ANOVA on the mean RTs revealed a main effect of TargetType, *F*(1, 38) = 78.70, *MS_e_* = 2,370, *p* < 0.001, *η_p_*^2^ = 0.67, indicating faster responses in the arrow condition (531 ms) than in the eye condition (600 ms). More importantly, TargetType and Congruency interacted, *F*(1, 38) = 13.28, *MS_e_* = 252, *p* < 0.001, *η_p_*^2^ = 0.26. While there was a reverse congruency effect in the gaze condition (−14 ms), no congruency effect was found in the eye condition (4 ms). No main effect of Congruency was found, *F*(1, 38) = 1.86, *MS_e_* = 555, *p* = 0.18, *η_p_*^2^ = 0.05.

**Table 1:** Means error rates (percentage incorrect) and within-subjects standard errors in Experiments 1–3.

Target type	Congruent		Incongruent	
	*M*	SE	*M*	SE
*Experiment 1*				
Eye	2.3	0.4	4.2	0.4
Arrow	2.4	0.3	3.9	0.4
*Experiment 2*				
Eye	2.3	0.3	3.0	0.4
Symbol	2.3	0.3	3.3	0.3
*Experiment 3*				
Eye	2.2	0.4	3.3	0.4
Symbol	1.7	0.3	3.1	0.3
Arrow	1.9	0.4	5.3	0.4

A similar analysis on the error rates showed a main effect of Congruency, *F*(1, 38) = 12.93, *MS_e_* = 8, *p* < 0.001, *η_p_*^2^ = 0.25, indicating higher accuracy in the congruent condition (2.4% error rate) than in the incongruent condition (4.0% error rate). Neither TargetType nor its interaction with Congruency reached significance, *F*(1, 38) < 1 in both cases.

As in Marotta et al. (2018), we found different patterns of data between the eye and arrow conditions. In both experiments, participants showed a significant congruency effect in the arrow condition (both RT and accuracy in Marotta et al., and accuracy in Experiment 1), and a significant inverse congruency effect in the RTs in the eye condition. However, unlike Marotta et al., in which the RT and accuracy results were in the same direction in the eye condition, the participants in Experiment 1 showed an inverse congruency effect in the RTs but a positive congruency effect in the error rates.^2^ Given that small differences in error rate can lead to large differences in response time (Pachella, 1974), and factors such as the response threshold level (Bogacz et al., 2010), the rate of information accumulation (Heitz, 2014), and motor execution processes (Spieser et al., 2017) can all affect the speed and accuracy of responses when participants are under speed pressure, it is unclear to what extent we can state with confidence that an inverse congruency effect was found in the eye condition of the present experiment.

To better understand the results in the eye condition, we calculated each participant’s balanced integration score (Liesefeld et al., 2015; Liesefeld and Janczyk, 2019), a measure that integrates speed and accuracy with equal weights (*Z*PC–*Z* $\bar{\mathrm{RT}}$ ). No difference was found between the congruent (−0.24) and incongruent (0.00) trials, *t*(39) = 0.86, *p* = 0.20, Cohen’s *d* = 0.14.

Why was there no congruency effect in the eye condition? Recall that the task was to respond to the direction indicated by the targets with reference to external space, not with reference to the location of the targets on the screen. From the participant’s perspective, location was a task irrelevant feature that could be ignored. It is possible that in the eye condition the low perceivability of the directional information induced a small attentional zoom, and this reduced the quality of the location representations of the target and the background rectangle. Consequently, no spatial congruency effect was found. We will discuss this more in the General discussion.

## Experiment 2

In Experiment 1, stimulus perceivability and the nature of stimulus type were intentionally confounded. In Experiment 2, we replaced the arrows with modified infinity symbols that resembled the eyes in perceivability but differed from the eyes in social significance (see Figure 1B, and the Supplementary material for the method). If similar patterns of data were found in the two conditions, this would indicate that stimulus perceivability likely played a key role in the results of Experiment 1.

### Results and discussion

Table 1 shows the error rates and Figure 2B shows the mean RTs. A repeated-measures ANOVA on the RTs revealed faster responses in the eye condition (577 ms) than in the symbol condition (596 ms), *F*(1, 38) = 6.34, *MS_e_* = 2,265, *p* = 0.02, *η_p_*^2^ = 0.14, and in the incongruent condition (583 ms) than in the congruent condition (590 ms), *F*(1, 38) = 5.57, *MS_e_* = 375, *p* = 0.02, *η_p_*^2^ = 0.13. TargetType and Congruency did not interact, *F* < 1.

Analysis on the accuracy data showed that the main effect of Congruency was just shy of significance, *F*(1, 38) = 3.66, *MS_e_* = 7, *p* = 0.06, *η_p_*^2^ = 0.09, with higher accuracy in the congruent condition (2.3% error rate) than in the incongruent condition (3.1% error rate). Neither TargetType nor its interaction with Congruency was reliable, *F* < 1 in both cases.

As in Experiment 1, the direction of the congruency effects in the eye condition differed between the RT and accuracy data. Importantly, a similar pattern of data was observed in the symbol condition. As infinity symbols can hardly be considered important in social communication, these results indicated that the nature of stimulus type was unlikely to be the main factor that contributed to the results in Experiment 1.

It should be noted that the above conclusion was based on the assumption that the infinity symbols used in Experiment 2 were not perceived as eyes. Admittedly, this assumption could be challenged because it was based on the responses from an independent group of participants, not on the responses from the actual participants of Experiment 2.^3^ It could also be argued that the stimulus perceivability in the eye and symbol conditions was not equivalent as responses were slower in the symbol condition than in the eye condition. Experiment 3 dressed these issues.

## Experiment 3

In Experiment 3, we varied the target stimuli (cartoon eyes, symbols, or two arrows) while controlling for stimulus perceivability (see Figure 1C). To prevent any carry-over effects that might affect the interpretation of results, we used a between-subjects design, with TargetType (eye, symbol, or arrow) as a between-subjects variable and Congruency (congruent or incongruent) as a within-subjects variable.

We also measured the participants’ perception of the targets in a naming task before they started the direction discrimination task. In the naming task, participants saw the targets (i.e., the same as those in the subsequent task) at the center of the screen, and they were required to respond, *via* keyboard input, with the first thing that came to mind. Upon completing the task, they proceeded to the direction discrimination task. If comparable congruency effects were found across the different groups, this would support the perceivability account as the primary interpretation of the results in the present study.

### Results and discussion

Most participants perceived the cartoon eyes as eyes (18/20), the nonsense symbols as non-eyes (18/20). All perceived the arrows as arrows or signs.

**Figure 3:**
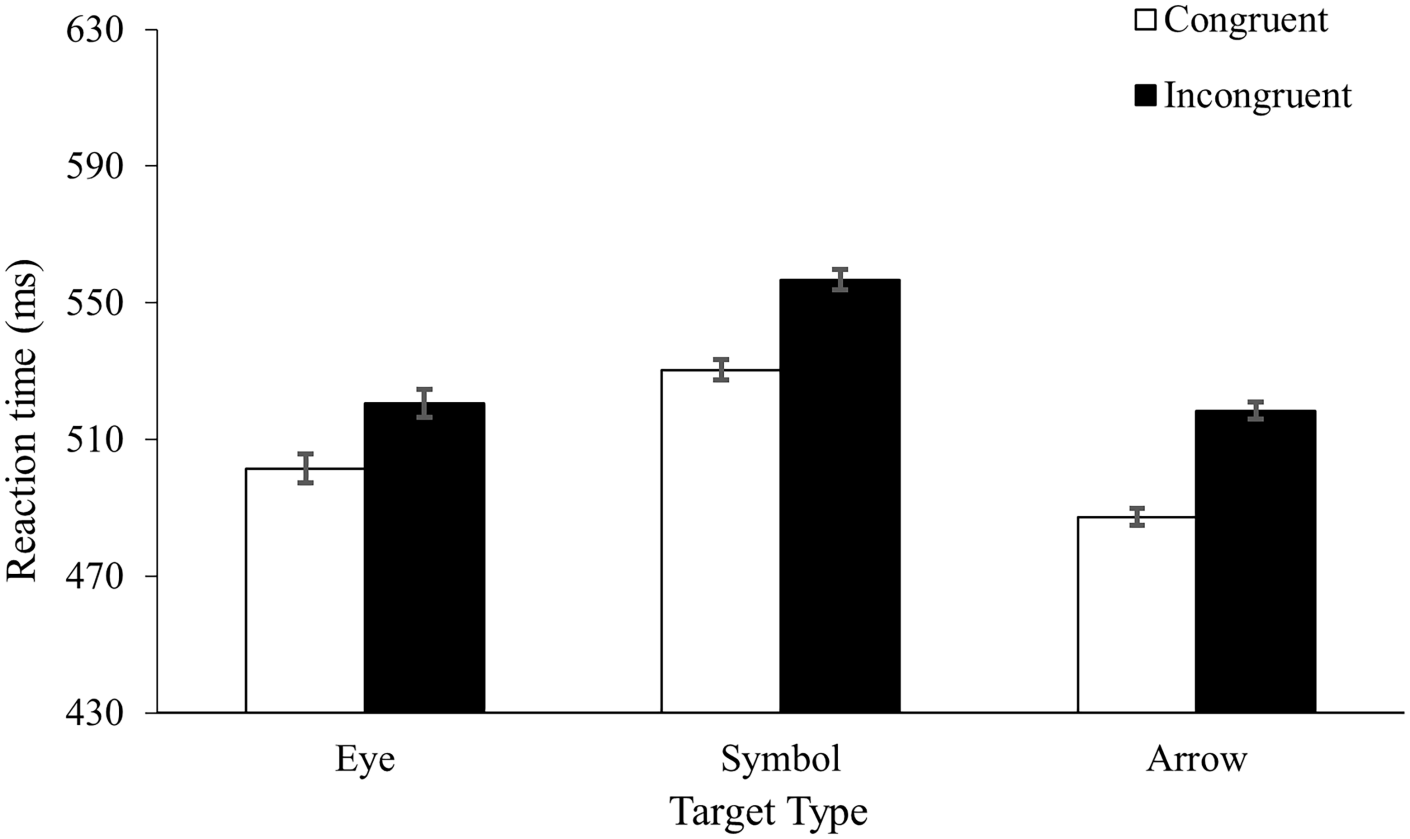
Results from Experiment 3.

Table 1 shows the error rates and Figure 3 shows the mean RTs. Two mixed ANOVAs, one on the mean RTs and the other on the error rates, found faster and more accurate responses in the congruent condition (504 ms with a 2.0% error rate) than in the incongruent condition (531 ms with a 4.0% error rate), *F*(1, 53) = 48.63, *MS_e_* = 426, *p* < 0.001, *η_p_*^2^ = 0.48, and *F*(1, 53) = 23.04, *MS_e_* = 5, *p* < 0.001, *η_p_*^2^ = 0.30, for the RT and accuracy, respectively. Importantly, the TargetType by Congruency interaction was not reliable in the RTs, *F* < 1. Although the interaction was close to significance in accuracy, *F*(2, 53) = 2.97, *MS_e_* = 5, *p* = 0.06, *η_p_*^2^ = 0.10, it was driven primarily by a numerically larger congruency effect in the arrow condition (a difference of 3.3% error rate) than in the eye condition (a difference of 1.2% error rate) or the symbol condition (a difference of 1.4% error rate). No main effect of TargetType was found in either RT (*p* = 0.26) or accuracy (*p* = 0.35).

The most important finding of the experiment was the comparable spatial congruency effects in the three groups. This result was important because it indicated that the different congruency effects between the eye and arrow conditions in Experiment 1 were unlikely to be caused by the nature of the stimuli. When the perceivability of the target sets became comparable in Experiment 3, the same pattern of data emerged, indicating that perceivability played a primary role in the results of the present study. Experiment 3 also provided additional evidence to the finding of Experiment 2. With the naming task and the use of a between-subjects design, we are reasonably confident that different groups of participants perceived the different sets of targets as being different in nature. Taken together, our data showed that stimulus perceivability rather than the nature of stimulus type played a key role in the processing of directional information in the present study.

## General discussion

We found no evidence that the encoding of the directional information by gaze and non-gaze stimuli differed when they had similar perceivability. In Experiment 1, the targets consisted of realistic-looking eyes or arrows, and the congruency effects differed between the two conditions. However, because stimulus type varied systematically with perceivability, the difference in results between the two conditions could be caused by either factor or both factors. In Experiment 2, the targets were eyes or infinity symbols. Despite different stimulus types, similar patterns of congruency effects were found in the two conditions. Experiment 3 further showed no difference in the spatial congruency effects across target types when stimulus perceivability was comparable.

Why were there no congruency effects in the eye and infinity symbol conditions in Experiments 1 and 2? We interpret these results in the framework of the zoom lens model of attention (Eriksen and St. James, 1986). According to the model, attention is like a zoom lens that can vary in size. When attention is zoomed out, the distribution of attentional resources is spread over a relatively large region, making processing less efficient. When attention is zoomed in, there is a greater concentration of resources in a smaller region, making processing more efficient. Moreover, the size of attentional zoom is typically triggered by task demand, with a more difficult task inducing a smaller attentional zoom. Previous research has shown that attentional zoom can affect the processing efficiency of the target and the degree of distractor interference (LaBerge and Brown, 1986; LaBerge et al., 1991; Chen and Chan, 2007; Chen and Cave, 2016). In the present study, as the directional information conveyed by the eyes and the infinity symbols was hard to perceive in Experiments 1 and 2, a small attentional zoom was required, and this in turn decreased the quality of the representation of the locations of the targets on the screen, resulting in the absence of the congruency effect.

Our study found no evidence that spatial congruency effects differed between the gaze and non-gaze stimuli when the stimuli had comparable perceivability in Experiment 3. This result is consistent with a number of previous studies, in which gaze and non-gaze stimuli were used as a spatial cue to direct participants’ attention to an upcoming target (e.g., Ristic et al., 2002; Kuhn and Benson, 2007; Tipples, 2008; Brignani et al., 2009; Marotta et al., 2013). Brignani et al. measured the participants’ event-related potentials (ERPs) triggered by a gaze or an arrow cue relative to an endogenous cue made of textures. The result most relevant to the present study was that no differences were found in the ERPs between the gaze and arrow cues. Similarly, Kuhn and Benson reported no difference in saccade latencies to a peripheral target regardless of whether a centrally located distractor was averted gaze or a directional arrow. Marotta et al. used gaze or an arrow cue to direct participants’ attention to one of two objects and found object-based attention of similar magnitude in the two conditions (but see Marotta et al., 2012).

However, despite the above studies that indicate a shared attentional system for the processing of gaze and non-gaze stimuli, there is evidence that gaze can guide attention in a different manner than non-gaze stimuli, especially when the direction of the gaze is counter-predictive to the location of a target. Downing et al. (2004), Experiment 2 showed participants a target letter preceded by a cue, which was either a pair of eyes or a tongue looking/pointing left or right. When the location indicated by the cue was four times less likely to be the location of the target, automatic orienting was triggered by the eyes but not by the tongue. Similar results were reported by Friesen et al. (2004), who also used a counter-predictive cue and found facilitation for targets appearing at the cued location when the cue was eyes, but not when it was an arrow. In both studies, the effect of the cue occurred only when the stimulus-onset-asynchrony (SOA) between the cue and the target was short (i.e., about 300 ms). These results indicate that gaze can automatically trigger attentional orienting, and that at short SOAs gaze can penetrate top-down attentional control better than non-gaze stimuli.

That the perception of gaze may be special in some way is also consistent with the findings of several physiological and neuroimaging studies. For example, there are cells in the macaque temporal cortex that respond selectively to the orientation of the head and gaze, but not to other objects such as food (Perrett et al., 1985). Monkeys with their superior temporal sulcus regions removed are impaired in tasks that require them to discriminate the direction of gaze (Campbell et al., 1990). Orienting by gaze and arrow cues activates different neural networks (Hietanen et al., 2006). Moreover, responses to the direction indicated by gaze and arrows elicited opposite effects in the later event-related potential components such as N2 and P3 (Marotta et al., 2019). It needs to be noted, however, that stimulus perceivability across the different types of targets was not controlled in the latter two studies.

In light of the seemingly inconsistent results described above, where does that leave us in terms of whether there is a special attentional system involved in the processing of gaze? One possibility is that such a system exists. However, because most previous studies in support of this view did not control stimulus perceivability, their results are subject to alternative interpretations, as we have demonstrated in the present study. We agree with Downing et al. (2004) that simple orienting effects may not highlight the unique properties of gaze processing. These properties, such as resistance to top-down attentional control, may require a more sensitive experimental paradigm to reveal. Another possibility is that in most simple orienting tasks such as those performed in a laboratory setting, the same system is involved in the processing of gaze and non-gaze stimuli. However, when a task becomes more complicated, such as when there is an urgent need to interpret the intention of the gaze or to make speeded counter-predictive attentional orienting, additional mechanisms in the brain are recruited to perform the task.

In summary, our results underscore the importance of controlling for stimulus perceivability when making inferences about the attentional mechanisms in the processing of gaze vs. non-gaze stimuli. While there is evidence for a special attentional system for gaze perception, the evidence may be more limited than has been assumed.

## Data availability statement

The raw data supporting the conclusions of this article can be found at https://osf.io/wy2d8/.

## Ethics statement

The studies involving human participants were reviewed and approved by Human Research Ethics Committee, the University of Canterbury. The participants provided their written informed consent to participate in this study.

## Author contributions

ZC generated the experimental ideas and programmed the Experiments 1 and 2. RHT conducted the Experiments 1 and 2, and analyzed the data with input from ZC. MSC programmed and conducted Experiment 1, and analyzed the data. All authors contributed to the article and approved the submitted version.

## Funding

This research was funded partly by the School of Psychology, Speech and Hearing, the University of Canterbury.

## Conflict of interest

The authors declare that the research was conducted in the absence of any commercial or financial relationships that could be construed as a potential conflict of interest.

## Publisher’s note

All claims expressed in this article are solely those of the authors and do not necessarily represent those of their affiliated organizations, or those of the publisher, the editors and the reviewers. Any product that may be evaluated in this article, or claim that may be made by its manufacturer, is not guaranteed or endorsed by the publisher.

## Footnotes

^1^We thank Andrea Marotta for providing us with the stimuli.

^2^Despite the differences in results, we do not consider our experiment as a failure to replicate Marotta et al. (2018). Although Experiment 1 was modeled after the experiment in Marotta et al., there were methodological differences that could lead to different results (e.g., the targets were smaller and closer to the fixation in our experiment than in theirs; and feedback was provided in their experiment but not in ours).

^3^We thank the reviewer for pointing this out.
