# Supplementary material for: Do gaze and non-gaze stimuli trigger different spatial interference effects? It depends on stimulus perceivability
Source: Front Psychol. 2022 Sep 13;13:801151. doi: 10.3389/fpsyg.2022.801151 (PMC9513585; doi:10.3389/fpsyg.2022.801151)
Supplement: Supplementary file 1 [file Data_Sheet_1.docx]

**Supplementary Information for “Do gaze and non-gaze stimuli trigger different spatial congruency effects? It depends on stimulus perceivability”**

*Zhe Chen, Rebecca H. Thomas, and Makayla S. Chen*

**Experiment 1**

**Method**

***Participants***

Forty students from the University of Canterbury participated in the experiment in exchange for course credit. The sample size was based on Marotta et al. (2018), who reported an effect size of *η*_p_^2^ = .54 for the interaction between stimulus type and congruency. For *α* = 0.05, a power analysis with G*Power 3.1 (Faul et al., 2009) showed that a sample size of 40 should provide us with over 95% power to detect the effect.

*Apparatus and Stimuli*. All the stimuli were presented against a black background. Each trial consisted of a white fixation cross (0.4° × 0.4°) and a pair of identical targets, which were either two eyes surrounded by flesh-colored eyelids looking left or right, or two black arrows pointing left or right. The two pairs of stimuli had the same overall size (2.7° × 0.9° in length and width). The targets were presented at the center of a grey rectangle (6.3° × 10.8° in length and width) whose center was 5.1° left or right of fixation.

***Design and Procedure***

Each trial started with a 1,000 ms fixation followed by the targets, which remained on the screen until response or after 2,000 ms had passed, whichever came first. In different blocks, either two eyes or two arrows would appear. They were equally likely to be on the left or right side of the screen, and were equally likely to look or point left or right. The location of the targets and the direction they indicated were congruent on half the trials (e.g., a target pointing left was on the left side of the screen) and incongruent on the other half (e.g., a target pointing left was on the right side of the screen). Thus, the experiment used a 2 × 2 repeated-measures design, with TargetType (eye vs. arrow) and Congruency (congruent vs. incongruent) as the two factors.

The task was to respond to the direction indicated by the targets. Participants pressed one of two labelled keys to respond “left” (the “z” key) or “right (the “m” key). No feedback was given during the experiment. Each participant completed two blocks. Half the participants started with the eye block, and the other half with the arrow block. Each block consisted of 128 experimental trials preceded by 16 practice trials, all having the same target type. Both speed and accuracy were emphasized.

**Data treatment**

The same data treatment criteria were used in all three experiments. Participants whose error rates were higher than 25% in any condition were excluded from analyses, and this resulted in the exclusion of one participant’s data. As in Marotta et al. (2018), trials faster than 200 ms or slower than 1300 ms were excluded, and this led to the loss of less than 1% of the trials in each experiment.

**Experiment 2**

**Method**

Experiment 2 used the same method as that of Experiment 1 except that the pair of arrows were replaced by two infinity symbols. To prevent the latter from being perceived as eyes, we changed the overall shape of the stimuli from ovals to rectangles while keeping the task relevant parts of the two stimulus sets (i.e., those that provided the directional information) as similar-looking as possible. Each infinity symbol had two parts, one colored dark brown and the other light gray, and each part was equally likely to be on the left or the right side of the stimulus. The task was to indicate whether the dark part was on the left side or the right side. Forty new participants took part in the experiment. Unfortunately, one participant’s data were lost due to computer malfunction.

To make sure that the infinity symbols would not be perceived as eyes, before we conducted Experiment 2, we approached 11 randomly selected naïve individuals on campus. Each was shown the pair of infinity symbols used in the experiment on a piece of paper, was asked to name the stimuli with the first thing that came to mind. (Note: the size of the stimuli was larger on the paper than that in the actual experiment.) None perceived the stimuli as eyes. Instead, the following names were given: hourglasses (4), egg timers (2), random shapes (2), tad poles (1), power plugs (1), and pebbles (1).

**Experiment 3**

**Method**

The method was the same as that in Experiment 1 except for the following changes. First, three new sets of targets were used in the direction discrimination task, one for each group of 20 participants. The targets were a pair of cartoon eyes, two nonsense symbols that we made by re-arranging the black and white components of the cartoon eyes, or two arrows with slender heads. Thus, the experiment used a 3 × 2 mixed design, with TargetType (eye, shape, or arrow) as a between-subjects variable and Congruency (congruent or incongruent) as a within-subjects variable. Second, to increase stimulus perceivability and the magnitude of the spatial Stroop effect, we increased the size of the targets and their eccentricity from fixation. All the three sets of targets had the same overall size of 4.1° × 1° in length and width. Each target stimulus subtended 1.6° × 1° in length and width, and the separation between the two items in any target set was 0.9°. The center of the rectangle was 7.5° left or right of fixation. Third, all stimuli were presented directly against a gray background. Participants completed a one-trial naming task before they proceeded to the direction discrimination task. In the naming task, they saw a pair of stimuli indicated the left direction at the center of the screen. The stimuli were the same as the targets in the subsequent direction discrimination task. Participants were required to type, in a horizontal box below the stimuli, the first thing that came to their mind. Once they completed the naming task, they proceeded to the direction discrimination task. Finally, each participant completed 256 trials. The experiment took about 20 minutes to finish.

**Results**

We conducted two sets of statistical analyses on the data for the direction discrimination task, one with four participants’ data excluded (two for perceiving the eyes as non-eyes and the other two for perceiving the symbols as eyes), and the other with no participants’ data excluded. Similar results were found in both cases.

**References**

Faul, F., Erdfelder, E., Buchner, A., & Lang, A. (2009). Statistical power analyses using GPower 3.1: Tests for correlation and regression analyses. *Behavior Research Methods*, *41*(4), 1149-1160. <https://doi.org/10.3758/BRM.41.4.1149>

Marotta, A., Román-Caballero, R., & Lupiañez, J. (2018). Arrows don't look at you: Qualitatively different attentional mechanisms triggered by gaze and arrows. *Psychonomic Bulletin & Review*, *25*(6), 2254-2259. https://doi.org/10.3758/s13423-018-1457-2
